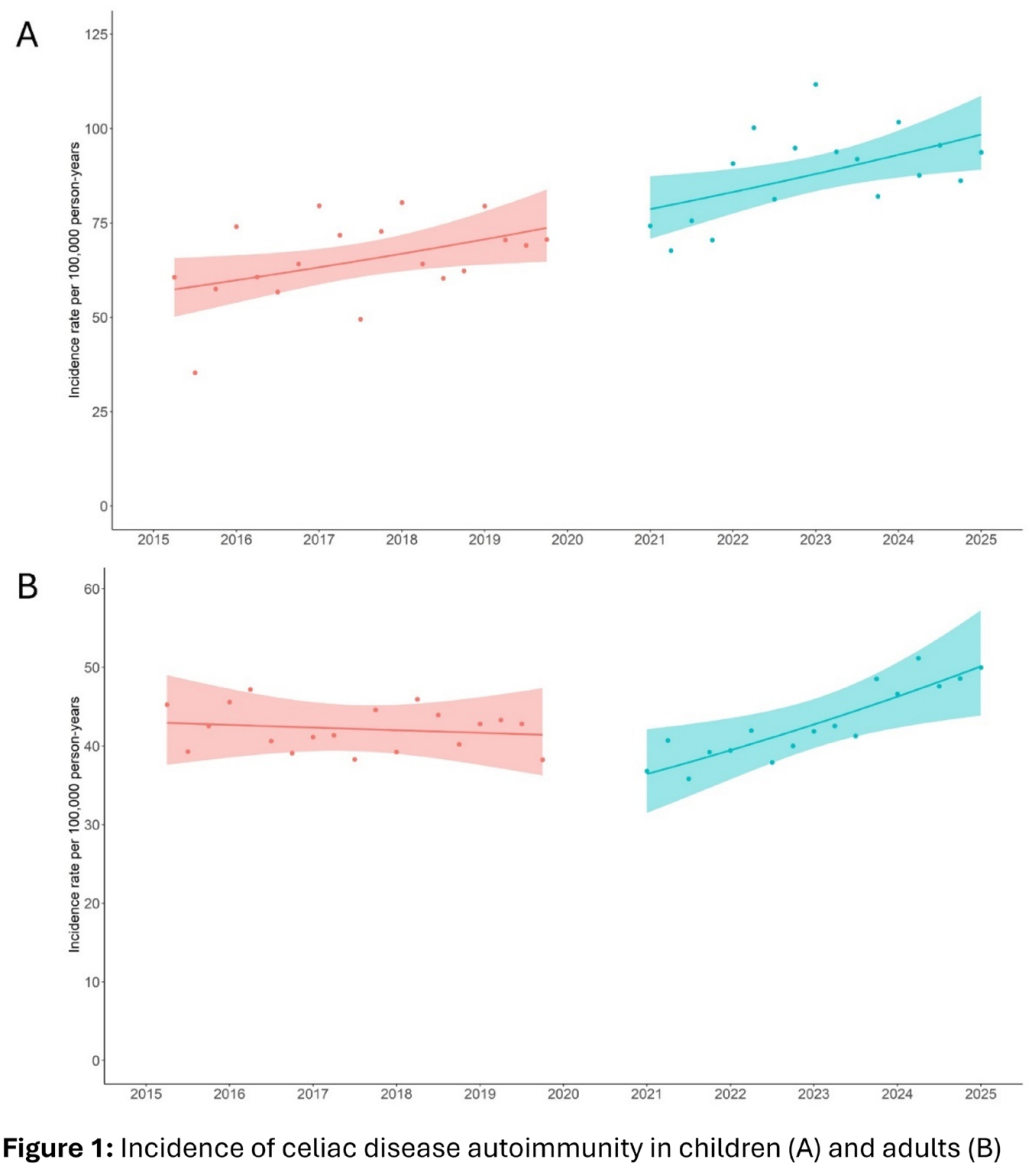# Poster Session I - A80 SIMILAR AND DIFFERENT IN A POST-PANDEMIC ERA: TRENDS IN THE INCIDENCE OF CELIAC DISEASE AUTOIMMUNITY IN ALBERTA

**DOI:** 10.1093/jcag/gwaf042.080

**Published:** 2026-02-13

**Authors:** J A King, S Coward, J Godley, A Metcalfe, P Ronksley, G G Kaplan, T Williamson

**Affiliations:** Community Health Sciences, University of Calgary, Calgary, AB, Canada; University of Calgary, Calgary, AB, Canada; Community Health Sciences, University of Calgary, Calgary, AB, Canada; Community Health Sciences, University of Calgary, Calgary, AB, Canada; Community Health Sciences, University of Calgary, Calgary, AB, Canada; Community Health Sciences, University of Calgary, Calgary, AB, Canada; Community Health Sciences, University of Calgary, Calgary, AB, Canada

## Abstract

**Background:**

In Alberta, Canada, rates of testing for celiac disease (CeD) based on tissue transglutaminase antibodies (tTG-IgA) remained stable from 2012 to 2019; however, incidence of CeD autoimmunity increased by 6% per year from 31.5 per 100,000 person-years in 2015 to 40.3 per 100,000 person-years in 2019.

**Aims:**

To understand if and how trends in the incidence of CeD autoimmunity have been affected from potential delays in identifying chronic diseases.

**Methods:**

All records for tTG-IgA were identified in Alberta between April 1, 2012 and March 31, 2025 from a province-wide laboratory database. Incident cases of CeD autoimmunity were defined as unique individuals newly positive for tTG-IgA between April 1, 2015 and March 31, 2025. Rates of CeD autoimmunity were analyzed by quarter to capture variation within years while balancing stability of estimates. Trends for two time periods—April 2015 to December 2019 (pre-pandemic) and January 2021 to March 2025 (post-pandemic)—were modelled using negative binomial regression while incorporating the population at-risk as an offset term. Average percentage changes and 95% confidence intervals (CI) across quarters in the two time periods were estimated from the model.

**Results:**

Observed incidence of CeD autoimmunity in children ranged between 35.3 (July–September 2020) to 111.7 (January–March 2023) per 100,000 person-years (Figure 1A). Although incidence rates were higher in the post-pandemic period, there was no significant difference in the trend across time (*p*=0.947). On average, pediatric incidence increased per quarter by 1.40% (95% CI: 0.26, 2.56) prior to the pandemic versus 1.46% (95% CI: –0.59, 3.56) in the post-pandemic period. Incidence of CeD autoimmunity in adults ranged from 21.1 (April-June 2020) to 51.2 (April-June 2024) per 100,000 person-years (Figure 1B). There was a statistically significant change in the trend across time between the two periods (*p*<0.001). On average, adult incidence was stable across quarters prior to the pandemic (–0.20, 95% CI: –0.66, 0.27) while it increased by 2.01% (95% CI: 1.15, 2.88) post-pandemic.

**Conclusions:**

Rates of adult CeD autoimmunity in the post-pandemic era were similar to prior estimates; however, trends showed an increased incidence in recent years, which could indicate an eventual ‘catching up’ of delayed diagnoses among adults. Regardless, further surveillance of CeD diagnoses in the coming years will establish whether a continued increase of pediatric CeD autoimmunity observed in the pre-pandemic period will persist, or if a plateau is on the horizon that has been reported in other settings.

**Funding Agencies:**

CIHRJames A King received the Canadian Institutes of Health Research (CIHR) Canada Graduate Scholarships Doctoral Award and TRIANGLE (TRaIning A New generation of researchers in Gastroenterology and LivEr) PhD Award to support his graduate program. This study was also supported by the Alberta Strategy for Patient Oriented Research Support Unit (AbSPORU) which is funded by CIHR, Alberta Innovates, University Hospital Foundation, University of Alberta, University of Calgary and Alberta Health Services.